# Pain Experienced during Various Dental Procedures: Clinical Trial Comparing the Use of Traditional Syringes with the Controlled-Flow Delivery Dentapen^®^ Technique

**DOI:** 10.3390/medicina57121335

**Published:** 2021-12-07

**Authors:** Erick Rafael Fernández-Castellano, Leticia Alejandra Blanco-Antona, Purificación Vicente-Galindo, Víctor Amor-Esteban, Javier Flores-Fraile

**Affiliations:** 1 Department of Surgery, University of Salamanca, 37007 Salamanca, Spain; kcire008@yahoo.es (E.R.F.-C.); lblanco@usal.es (L.A.B.-A.); 2 Salamanca Biomedical Research Institute (IBSAL), 37007 Salamanca, Spain; purivg@usal.es; 3 Department of Biostatistics, University of Salamanca, 37007 Salamanca, Spain; vamor@usal.es

**Keywords:** local anaesthesia, pulse rate, computer-delivered anaesthesia, pain

## Abstract

*Background and Objectives*: Currently, one of the most discouraging aspects for many patients undergoing dental procedures is the administration of anaesthesia. Consequently, there is a constant search for new techniques to avoid the invasive and painful nature of the injection. A new motorised syringe system (Dentapen^®^) has recently been developed, standing out for its convenience and ease of use. *Material and Methods:* Randomised, controlled, single-blind, and single-centre study including 178 voluntary adult participants aged between 18 and 90 years. Individuals were randomly assigned using a randomised table. Patients were asked to rate the level of pain experienced during the injections, using a 10-point visual analogue scale (VAS). The following data were recorded: pain index, heart rate, blood pressure, and saturation, both before and after anaesthesia. *Results:* Of the total 178 participants, 87 participants (48.9%) were men and 91 (51.1%) were women. The first variable to be assessed was the pain experienced by patients when anaesthetised with a syringe, obtaining a mean value of 2.63 ± 1.86 on the VAS with the conventional syringe and 1.06 ± 1.28 with the Dentapen^®^ syringe, showing statistically significant differences (*p*-value < 0.01). When stratifying, based on the procedure that was undertaken, differences were also significant for all treatments (*p*-value < 0.01) except for endodontics, where differences were likely to be significant (*p*-value = 0.02). *Conclusions:* In conclusion, from a clinical standpoint, the Dentapen^®^ syringe is a valid alternative to traditional infiltration syringes, causing minimum pain with the injection.

## 1. Introduction

At present, the administration of anaesthesia is one of the most discouraging aspects for most patients undergoing dental procedures, causing many of them [1,2,3,4] to feel anxious and uncomfortable. This situation can hinder our therapeutic efforts and cause avoidable discomfort on the patients [1,5,6,7]. Pain during anaesthesia can be caused by the needle itself or by the injection of the anaesthetic substance [1,5,8,9,10,11], aggravated by the patient’s anxiety or fears [1,6,11]. Although the aim of local anaesthesia is to eliminate pain during dental procedures, the apprehension associated with needles and punctures is often considered reason enough for not visiting the dentist [3,12,13,14].

Grace et al. stated, in their studies conducted on adolescents and young adults from different countries (Belfast (Northern Ireland), Helsinki (Finland), Jyväskylä (Finland), Dubai (UAE), Dunedin (New Zealand), and Singapore), that dental phobias affected 5–15% of them, and that 11–26% experienced high levels of dental fear and anxiety [3,15,16,17,18].

In a cross-sectional study on 970 children between the ages of 5 and 12 years, Colares et al. found a prevalence of dental fear and anxiety of 14.4% [19]. The greatest fear was associated with injections [20].

A study on the prevalence of the clinical consequences of untreated tooth decay and its connection with dental fear found that children with fear were 2.05 times more at risk of tooth decay than those with little or no fear [21].

The premise that justifies our study is that the slow injection of anaesthetics at low pressure appears to reduce pain and discomfort during dental anaesthesia. An accurate control of flow and pressure of the injection can therefore mitigate the pain experienced by these patients. Primosch and Brooks revealed that injecting 0.3 mL of local anaesthetic solution at a slow speed with constant flow (161 s/mL) is less painful than with a quicker infiltration (29 s/mL). Other authors stated that, to minimise pain and anxiety, it is important for dentists to start injecting the anaesthetic at a pressure of under 306 mm Hg [4]. Consequently, there is a constant search for new techniques looking to avoid the invasive and often painful nature of the anaesthetic injection required for dental treatments, making it a more pleasant and less distressing experience for patients [10]. Even though there are no techniques available that can totally replace conventional local anaesthesia, some alternatives have been developed that are effective in a limited range of procedures [6]. In 1997, a new method for the administration of anaesthetics was launched: the computer-controlled local anaesthesia delivery system (CCLADS). After 2006, the single tooth anaesthesia system (STA) (Milestone Scientific, Inc. Livingston, NJ, USA) was also introduced [22].

A new, cableless, motorised syringe system (Dentapen^®^) has recently appeared that stands out for its convenience and ease of use, and that currently does not require specific training. It has several injection settings, allowing it to be held like a syringe or pen, and is compatible with all anaesthetic needles and cartridges from all brands.

The aim of this study was to compare the pain experienced by patients during local anaesthetic injections in different areas and procedures, using both the traditional syringe and the controlled flow technique with the Dentapen^®^ system.

## 2. Materials and Methods

We conducted a randomised, controlled, single-blind, and single-centre study following the Declaration of Helsinki guidelines and with the approval of the review board of the Ethics Committee of the University of Salamanca (Protocol number 542/2020). It comprised a single operator and 178 adult volunteers requiring some sort of dental treatment aged between 18 and 90 years, 87 of which (48.9%) were men and 91 (51.1%) of which were women. The study was carried out at the Dental Clinic of the University of Salamanca.

Treatment for each patient was assigned by a randomization list, automatically generated prior to the start of the study, in which the treatment material was determined. In the case of bilateral treatments, treatment was assigned to one side or the other, according to a supplementary randomization list. The patient randomization was performed in order to avoid sex bias; thus, the final numbers of men and women in each group were not significantly different, to comply with the blind aspect of the study—participants were not informed of the kind of syringe that was to be used. Patients were also asked to don a mask over their eyes, which remained closed throughout the procedure so as not to see the syringe. The sound of the Dentapen^®^ syringe motor was camouflaged with background music, together with the suction system of the dental chair.

An average of 1.8 ± 1.9 interventions that required anaesthesia were undertaken on each patient up to a total sample of 287 observations (51% in men and 49% in women). The distribution based on the type of procedure was as follows: 119 extractions, 66 fillings, 55 root canal treatments and root canal retreatments, 27 surgeries where a total of 11 implants were performed, 2 atraumatic and 1 traumatic maxillary sinus lift, 1 lip-repositioning surgery, 2 vestibuloplasties, 3 horizontal bone regenerations, 2 crown enlargements, 2 free gingival grafts, and 20 prepared tooth stumps. A Dentapen^®^ syringe was used in 54.7% of the procedures and a conventional syringe was used in the remaining 45.3%.

### 2.1. Injection with Dentapen^®^ (Septodont, Switzerland) and the Conventional Three-Ring Syringe (Asa Dental, Italy)

Each infiltration, irrespective of the type of syringe used, was preceded by the application of a spray containing lidocaine and cetrimonium bromide (Xilonor Spray, Septodont). After 3 min, the infiltration was carried out, with 1.8 mL of lidocaine 2% and 1: 80,000 (Xilonibsa; Inibsa, Lliçà de Vall, Spain). In the vestibular and lingual areas with Dentapen^®^, the green LED speed setting was used, together with the blue ramp-up. For the lower nerve-trunk blocks, however, the Akinosi technique was used, with the purple LED speed setting, together with the blue ramp-up mode. In palatine areas, the purple intra-ligamentary mode was used. Around 4–5 s after the puncture, the needle was further inserted apically, and the activation button was pressed twice to activate the self-suction mode and verify whether blood entered the cartridge or not. Patients were asked to rate the level of pain felt during the injection, using a 10-point visual analogue scale (VAS) that went from: 0–2—no pain, 2–4—moderate pain, 4–6—severe pain, 6–8—extreme pain, and 8–10—unbearable pain. In all cases, patients were placed in a supine position with the head tilted back. Variables, such as blood pressure, measured using a sphygmomanometer (Watch BP home, Switzerland), and saturation and heart rate, using a pulse oximeter (Contec Medical Systems, China), were controlled both before and after the anaesthetic injection. The needle used for the infiltrative techniques in the palatine, vestibular, and lingual areas was the Medicaline 30G short 0.3 mm × 25 mm needle, while the Medicaline 27G long 0.4 mm × 38 mm needle was used for the nerve trunks.

### 2.2. Inclusion Criteria

Healthy patients, aged between 18 and 90 years, who required any type of dental treatment that entailed the administration of an anaesthetic. All patients agreed to participate and signed the necessary informed consent form.

### 2.3. Exclusion Criteria

Patients with an ASA III and ASA IV classification, with gumboils, a history of psychiatric illness and/or allergies to lidocaine. Pregnant patients. Patients with drug and alcohol issues, or who had recently suffered a heart attack. Patients with respiratory issues in the last 14 days. Patients with COVID-19 or who were in quarantine.

### 2.4. Statistical Analysis

We evaluated the mean reported pain levels measured using the VAS (ranging from 0 (no pain), to 10 (unbearable pain)), based on the type of syringe used, the type of treatment, and the area of injection. We analysed gender-related differences, and whether there were any changes in blood pressure, saturation, or heart rate after anaesthesia with both syringes. To do so, results were expressed as means and standard deviations ($\bar{X}$ ± SD) and were analysed using a *t*-test—a statistical deductive tool that evaluates the differences in means between two groups through statistical hypothesis testing. This allowed us to determine, through the data of our sample, if the differences that were found can be generalized to the population, and to verify with a high level of confidence if the Dentapen^®^ syringe induces less pain than the conventional syringe. Similarly, we used an analysis of variance to prove the hypothesis that the means in more than two groups are the same. All analyses and data visualisations were carried out using SPSS 25.0 software (Endicott, NY, USA) and Microsoft Excel 16.46 (Redmond, WA, USA).

## 3. Results

A Dentapen^®^ syringe was used in 54.7% of the procedures and a conventional syringe was used in the remaining 45.3%. Regarding the area where the syringe was inserted, the most frequent was the vestibular area (50.9%), followed by the palatine (25.4%), the trigone (20.6%), and the lingual area (3.1%). This information can be found in Table 1.

**Figure 1:**
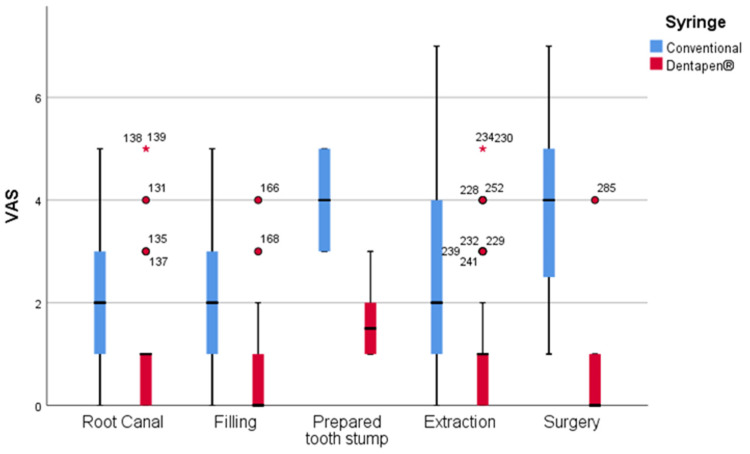
VAS (visual analogue scale) based on the type of treatment and the type of syringe used. BOX-PLOT VAS (visual analogue scale) based on the type of treatment and the type of syringe used.

Firstly, we assessed the pain experienced by patients (VAS scale) based on the type of syringe used, showing highly significant differences (*p*-value < 0.01), with lower reported pain when using the Dentapen^®^ syringe, with a mean value of 1.06 ± 1.28 points, compared with the results obtained in procedures where the conventional syringe was used, with 2.63 ± 1.86 mean points. These differences persisted when stratifying by the type of treatment, with highly significant differences (*p*-value < 0.01), except for in endodontics, where differences were likely to be significant (*p*-value = 0.02) (see Table 2 and Figure 1).

Secondly, we evaluated whether there were differences in the pain felt by patients based on the area of injection—palatine, trigone, vestibular, or lingual—where we found significant differences overall (*p*-value = 0.000). The lowest pain levels corresponded to the vestibular area (1.21 ± 1.49), which showed statistically significant differences when compared with the remaining areas: 2.49 ± 2.01 in the palatine area (*p*-value = 0.000), 2.08 ± 1.48 in the trigone area (*p*-value = 0.000) 2.06 in the lingual area (*p*-value = 0.000). (Table 3).

Furthermore, these differences increased when considering the use of a conventional syringe exclusively. In this case, and in addition to the same overall differences found in our previous analysis, we also identified differences when comparing the trigone area with the palatine (*p*-value = 0.001) and lingual areas (*p*-value = 0.031). By studying the sample values, we can observe that pain is more stable and notably lower when using the Dentapen^®^ syringe, with highly significant differences in all areas. This information is presented based on the type of syringe used in Table 4.

**Figure 2:**
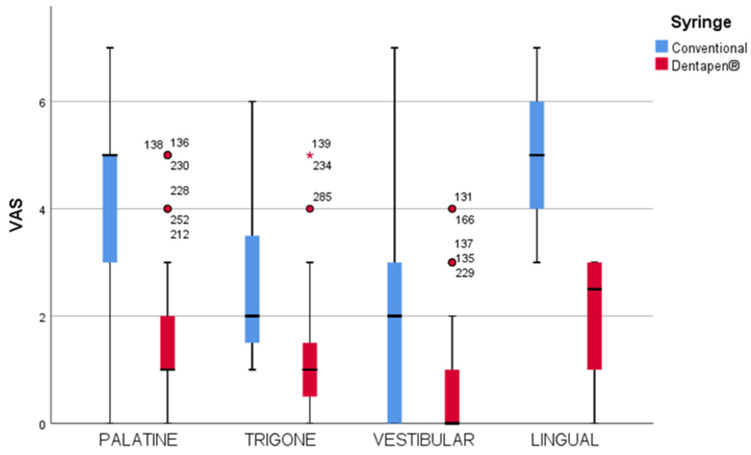
VAS comparison based on the area of injection and the type of syringe used. BOX-PLOT. VAS comparison based on the area of injection and the type of syringe used.

Figure 2 shows that, as was previously described, the pain experienced by patients was notably lower in all areas when using the Dentapen^®^ (red) syringe. Moreover, and in addition to offering higher stability, the differences based on the area of injection were also lower in this group. In contrast, both the pain and the differences by area sharply increased when using the conventional syringe (blue). It is worth mentioning that, with both syringes, patients felt less pain in the vestibular area, with significant differences in relation to the other areas.

**Figure 3:**
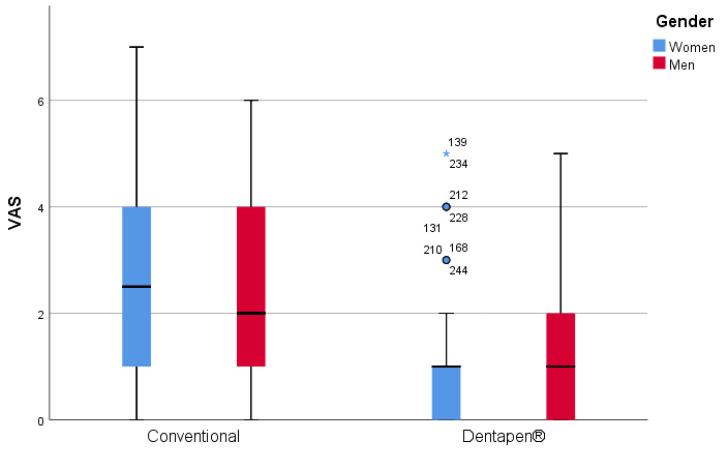
VAS comparison by gender. BOX-PLOT. VAS comparison and statistical significance by gender.

Thirdly, we studied the differences in the VAS score by gender (see Table 5 and Figure 3). The mean values were higher in women (1.86 ± 1.84) compared with men (1.68 ± 1.67), although these differences were not statistically significant (*p*-value = 0.518) globally, nor when stratifying by the type of syringe, area of injection, or type of treatment.

Additionally, we compared the need for several cartridges of anaesthesia between the two syringe types. In the entire sample, we had 196 observations of the placement of 1 cartridge, 82 observations of 2 cartridges, 5 of 3 cartridges (4 conventional and 1 Dentapen^®^) and 4 of 4 cartridges (2 conventional and 2 Dentapen^®^). No statistically significant differences were found (*p*-value = 0.0332).

Lastly, we analysed the differences in blood pressure, saturation, or heart rate after the administration of the anaesthetics with both syringes. For this analysis, we studied the information for each type of syringe separately, and we observed the changes of the different variables (see Table 6).

Heart rate: We observed a reduction in heart rate with both syringes, with greater variation in patients whose treatment involved the use of a conventional syringe (*p*-value = 0.044).

High blood pressure: In both cases, we observed a small reduction with statistical significance, which was slightly higher with the conventional syringe (*p*-value = 0.017) than with Dentapen^®^ (*p*-value = 0.049).

Low blood pressure: In both groups, a small reduction with statistical significance was observed, which was slightly higher with the Dentapen^®^ syringe (*p*-value = 0.016) than with the conventional syringe (*p*-value = 0.035).

Saturation: Saturation increased with both syringes, though this variation was only significant with Dentapen^®^ (*p*-value = 0.018).

When evaluating the differences in the variables mentioned above, based on the type of syringe, we found no statistical significance in the “before” and “after” values. We can therefore conclude that the values measured before the procedure do not vary based on the knowledge of the type of syringe that will be used.

## 4. Discussion

We evaluated patient-reported pain levels experienced during the administration of anaesthetics for dental procedures, comparing the results of using conventional syringes and the Dentapen^®^ system for local anaesthesia. Secondarily, we considered the variations in parameters such as heart rate, blood pressure, and saturation, before and after the injection.

The design chosen for this work was that of a randomised clinical trial with two groups. Although similar studies opted for a split-mouth approach, with the advantage that individuals can act as their own control, we decided against it due to the difficulty of recruiting patients with a need for similar dental interventions in both sides of the arch. Furthermore, the classification of the pain level of the second anaesthetic injection can be less reliable with these designs, given the influence of the first [4].

In this study, pain perceived by patients was notably lower in all the areas where the Dentapen^®^ syringe was used, which, in addition, offered greater stability. Regarding the use of conventional syringes, we observed a sharp increase in patient-reported pain, with differences based on the area of injection. Patients felt less pain in the vestibular area with both syringes, with significant differences compared with the other localisations. This concurs with the findings of Campanella et al. [23], Garret-Bernardin et al. [3], Grace et al. [15], and Mohammad et al. [22], who reported significantly lower pain levels with the STA injection compared with conventional local anaesthesia. However, these studies did not specify if the traditional anaesthetic procedure consisted of local infiltration or nerve block, the type of anaesthetic used, the techniques used, or if a local anaesthetic was applied before the insertion of the needle.

CCLAD systems can reduce injection flow to a specific, fixed pressure, regardless of the variation in tissue resistance [24]. This effect results in a controlled, effective, and less unpleasant injection, even in elastic tissues such as the palate or the periodontal ligament. Maintaining an ideal flow of anaesthetic solution is probably the most important factor needed to guarantee a controlled injection [25]. Our results appear to support this statement, given that the majority of patients mentioned mild pain. It is also worth highlighting that all the injections in this study were administered with a similar flow speed and with the same needle setting and local anaesthetic solution.

Overall, we found no significant differences in terms of patient-reported pain by gender. Our results when taking into account the type of syringe, the area of injection, and the type of treatment concurred with previous findings, such as in the studies by Gibson et al. and Allen et al. [9]. Similarly, there were no significant differences in terms of blood pressure, heart rate, or saturation variables before and after the injection. It seems that these variables do not vary prior to the intervention, irrespective of whether the patient discovers the type of syringe used or not. This differs from the results reported by San Martín-López et al. and Annelyse Garret-Bernardin et al., who found a greater increase in heart rate after the injection with the conventional syringe [3].

A variable of potential interest that could not be included in our analysis was the anxiety experienced by patients. Notwithstanding, not all previous studies on the matter had analysed it, presumably as a result of several authors, such as Tahmassebi et al. [26] and Campanella et al. [23], concluding that anxiety levels are independent from the anaesthetic device used. A recently published report also concluded that it was unnecessary to provide patients with a detailed explication on the CCLAD system, as doing so did not reduce their anxiety [1]. We therefore considered that, even if a new clinical trial were conducted including anxiety as a factor, results would not vary significantly.

## 5. Conclusions

In conclusion, from a clinical perspective, the Dentapen^®^ syringe is a valid alternative to conventional infiltration syringes, causing minimum pain with the injection. Regarding clinical discomfort, the use of the Dentapen^®^ syringe provides a deep anaesthetic effect, which we achieved in the different dental procedures undertaken for this study, thereby increasing patient satisfaction.

## Figures and Tables

**Table 1:** Frequency distribution table based on the area of injection and the type of treatment.

Treatment	Palatine	Trigone	Vestibular	Lingual	Total
Root canal	14	10	31	0	55
Filling	1	19	45	1	66
Prepared tooth stump	10	0	10	0	20
Extraction	39	29	45	6	119
Surgery	9	1	15	2	27

**Table 2:** VAS (visual analogue scale) comparison and statistical significance, based on type of treatment and the type of syringe used.

Treatment	Syringe	Sample	Average	SD	*p*-Value
Global	Conventional	130	2.63	1.86	0.000
	Dentapen^®^	157	1.06	1.28	
Surgery	Conventional	15	3.93	2.02	0.000
	Dentapen^®^	12	0.58	1.17	
Root canal	Conventional	24	2.00	1.47	0.020
	Dentapen^®^	31	1.16	1.61	
Extraction	Conventional	54	2.57	2.04	0.000
	Dentapen^®^	65	1.12	1.33	
Filling	Conventional	33	2.42	1.50	0.000
	Dentapen^®^	33	0.76	1.01	
Prepared tooth stump	Conventional	4	4.00	1.16	0.002
	Dentapen^®^	16	1.56	0.63	

**Table 3:** VAS comparison and statistical significance based on the area of injection.

Area 1	Area 2	Sample	Mean	SD	*p*-Value
Vestibular	Palatine	73	2.49	2.01	0.000
*n* = 146; 1.21 ± 1.49	Trigone	59	2.08	1.48	0.000
	Lingual	9	3.00	2.06	0.004
Palatine	Trigone	59	2.08	1.48	0.436
	Lingual	9	3.00	2.06	0.429
Trigone	Lingual	9	3.00	2.06	0.173

**Table 4:** VAS comparison and statistical significance based on the area of injection and the type of syringe used.

**Conventional Syringe**					
Area 1	Area 2	Sample	Average	SD	*p*-Value
VESTIBULAR	Palatine	27	4.07	1.88	0.000
*n* = 64; 1.97 ± 1.73	Trigone	36	2.53	1.29	0.049
	Lingual	3	5.00	2.00	0.023
Palatine	Trigone				0.001
	Lingual				0.480
Trigone	Lingual				0.031
**Dentapen^®^ Syringe**					
Area 1	Area 2	Sample	Average	SD	*p*-Value
Vestibular	Palatine	46	1.57	1.42	0.000
*n* = 82; 0.61 ± 0.92	Trigone	23	1.39	1.50	0.004
	Lingual	6	2.00	1.27	0.006
Palatine	Trigone				0.455
	Lingual				0.313
Trigone	Lingual				0.215

**Table 5:** VAS comparison and statistical significance by gender.

Syringe	Gender	Sample	Average	SD	*p*-Value
	Women	142	1.86	1.84	0.518
	Men	145	1.68	1.67	
Conventional	Women	66	2.83	1.88	0.233
	Men	64	2.42	1.84	
Dentapen^®^	Women	76	1.01	1.31	0.431
	Men	81	1.10	1.25	

**Table 6:** Heart rate, systolic and diastolic blood pressure, and saturation comparison, plus statistical significance after the use of conventional and Dentapen^®^ syringes.

Variable	Syringe	Time	Sample	Average	SD	*p*-Value
Heart rate	Conventional	Before	89	77.74	12.60	0.044
		After	89	75.92	9.69	
	Dentapen^®^	Before	89	78.38	10.66	0.085
		After	89	76.92	9.84	
Systolic blood pressure	Conventional	Before	89	130.33	14.95	0.017
		After	89	128.41	12.50	
	Dentapen^®^	Before	89	129.80	16.46	0.049
		After	89	128.00	13.20	
Diastolic blood pressure	Conventional	Before	89	78.86	8.82	0.035
		After	89	80.29	8.37	
	Dentapen^®^	Before	89	77.97	9.29	0.016
		After	89	79.44	7.70	
Saturation	Conventional	Before	89	96.50	1.89	0.069
		After	89	96.73	1.57	
	Dentapen^®^	Before	89	95.10	9.49	0.018
		After	89	95.39	9.42	

## Abbreviations

C-CLAD	Computer-controlled local anaesthetic delivery
STA	Single tooth anaesthesia
VAS	Visual analogue scale
